# A D-Band Dual-Polarized High-Gain LTCC-Based Reflectarray Antenna Using SIW Magnetoelectric-Dipole Elements

**DOI:** 10.3390/mi15121511

**Published:** 2024-12-20

**Authors:** Zhuo-Wei Miao

**Affiliations:** State Key Laboratory of Millimeter-Waves, School of Information Science and Engineering, Southeast University, Nanjing 210096, China; zwmiao@seu.edu.cn

**Keywords:** D-band, dual polarized, low-temperature co-fired-ceramic (LTCC) technology, magnetoelectric dipole, receiving/reradiating (R/R) principle, reflectarray (RA) antenna, substrate-integrated waveguide (SIW)

## Abstract

This paper presents a D-band dual linear-polarized wideband high-gain reflectarray (RA) antenna using low-temperature co-fired-ceramic (LTCC) technology. The proposed element comprises a dual-polarized magnetoelectric (ME) dipole and a multilayer slot-coupling substrate-integrated waveguide (SIW) phase-delay structure, which are organized in accordance with the receiving/reradiating (R/R) principle. The coverage of phase shifts for both orthogonal polarizations is set to be greater than 360 degrees by varying the length of the phase-delay structure. For verification, a D-band 1296-element RA prototype using the proposed unit cell is fabricated and measured in a THz chamber. The measured results show that the proposed RA achieves a peak gain of 32.25 and 33.03 dBi for the two orthogonal polarizations. The measured 3 dB gain bandwidths for the two orthogonal polarizations are 122–149 GHz (20%) and 123–149 GHz (19.3%), respectively.

## 1. Introduction

An exploration of higher mm-wave and sub-THz carrier frequencies is being conducted to accommodate the growing need for wireless communication bandwidths, such as the D-band (110–170 GHz) [1]. To compensate for its severe path loss, a wireless transceiver frontend operating in the D-band must work with enough effective isotropic radiated power (EIRP). Nevertheless, the challenge and expense of generating high-power radio frequency (RF) signals increase as the frequency rises, as do the power losses in substrates and interconnectors. Hence, both high gain and high efficiency are expected in the D-band antenna design [2].

Due to the desired features of high gain, high efficiency, low profile, and ease of fabrication, reflectarray (RA) antennas have been intensively studied for frequencies ranging from the microwave to THz bands over the last several decades based on different kinds of processes [3,4,5,6,7,8,9,10,11,12,13,14,15,16,17,18,19,20,21,22,23,24]. For planar RA antennas, several approaches have been proposed to improve the bandwidth performance of RAs, including stacked patch elements [9,10], multi-resonance elements [11,12,13], subwavelength elements [14,15], and coupling delay line (DL) elements [16,17,18,19]. Among these techniques, the DL-based method streamlines the design process by merely requiring consideration of the varying length of DLs for a 360° phase-shift range [20,21]. In addition, RAs have the capability to perform diverse operations in orthogonal polarization channels with low crosstalk if the unit cells are designed with independent phase modulation on orthogonal linear-polarized waves [20,21,22,23]. By flexibly manipulating the two orthogonal polarization components from a single-feed or dual-feed source with an RA, dual linear-polarized radiation, circular-polarized radiation, or even elliptically polarized radiation can be achieved. Therefore, there is a significant demand for D-band RAs that exhibit a high gain, a broad bandwidth, and polarization-switching ability simultaneously.

Due to rapid advancements in multilayer processes, low-temperature co-fired-ceramic (LTCC) technology offers a convenient and adaptable option for the production of D-band RA antennas [24]. However, to the best of the authors’ knowledge, very few studies have been reported in the literature for dual-polarized RA antennas based on LTCC technology operating at over 100 GHz. In this paper, a novel DL reflective element is proposed and used to implement a D-band dual linear-polarized (LP) wideband high-gain RA antenna based on LTCC technology. Considering its wide impedance bandwidth and stable radiation performance, a dual-polarized substrate-integrated waveguide (SIW) slot-coupled magnetoelectric (ME) dipole serves as the receiving/reradiating (R/R) part of this system. This kind of antenna applies the concept of a complementary antenna including an electric dipole and a magnetic dipole [25]. Under the R/R part, two different SIW slot-coupled transmission paths are employed to independently control the reflective phase responses for different polarizations. In the following sections, the element configuration and its performance are illustrated, and a D-band RA prototype is fabricated and measured for verification.

## 2. R/R ME-Dipole Element

### 2.1. Geometry and Operation Mechanism

**Figure 1:**
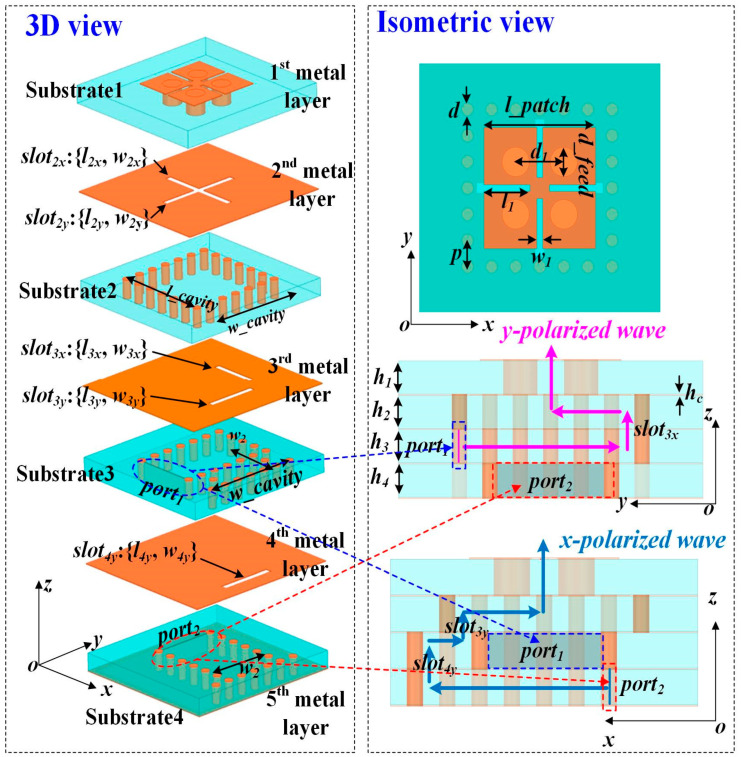
Configuration of the proposed dual-polarized SIW slot-coupled ME dipole as the receiving/reradiating element with two waveguide ports (Port 1 and Port 2).

Based on LTCC technology, the configuration of the R/R section in the proposed RA element is shown in Figure 1. The substrate used in this design is the Ferro A6-M, with a relative permittivity of 6 and a loss tangent of 0.002, which were measured at 100 GHz. As shown in Figure 1, Substrate 1 is where the dual-polarized ME-dipole structure fed by crossed slots is constructed. Four identical metallic patches on the top layer are connected to the ground through metallic vias. The apertures *slot*_2*x*_ and *slot*_2*y*_ are built on the second metal layer. They are employed to couple the D-band signals from Substrate 2 for *y-* and *x-*polarized radiation, respectively. For *y*-polarized radiation, through the aperture *slot*_3*x*_, D-band signals generated by Port 1 couple to Substrate 2, with the electric field lying on the *xoz-*plane. Port 1 is located in Substrate 3, acting as a source that excites the SIW transmission structure. When it comes to y-polarization, the apertures *slot*_3*y*_ and *slot*_4*y*_ are positioned along the *yoz-*plane, allowing the D-band signals generated from Port 2 in Substrate 4 to be coupled to Substrate 2. Port 2 is situated in Substrate 4 and is responsible for the excitation of the SIW transmission structure. In order to independently generate the two orthogonally polarized waves, it is necessary to obtain a high degree of isolation between the two polarizations in this design.

### 2.2. Radiation Performance

**Figure 2:**
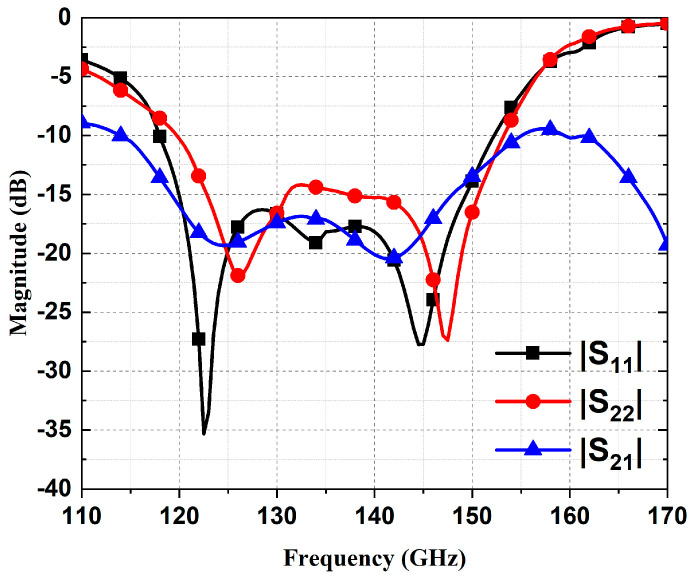
Simulated S-parameters of the receiving/reradiating ME-dipole element.

**Figure 3:**
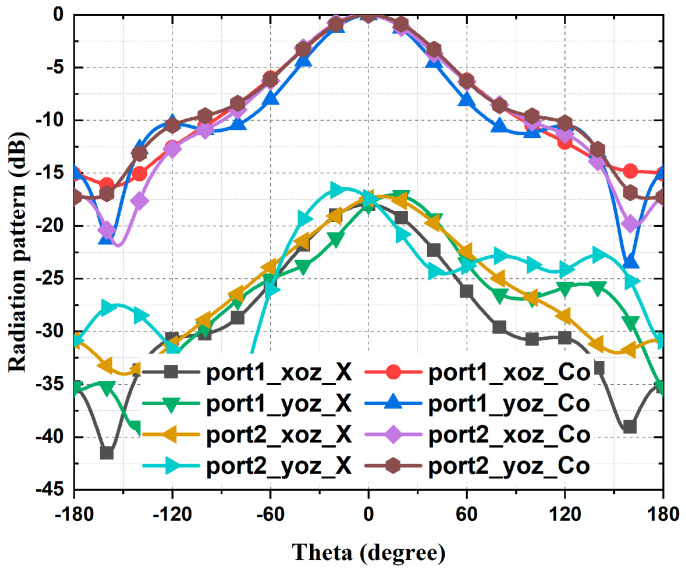
Simulated radiation patterns of the receiving/reradiating ME-dipole element.

The full-wave simulator Ansys HFSS is employed to adjust the geometries. The final values of the dimensions of the receiving/reradiating element are detailed in Table 1. The simulated S-parameters of the dual-polarized ME-dipole element are shown in Figure 2. The overlapping impedance bandwidth of the two polarizations for |S_11_| and |S_22_| smaller than −10 dB ranges from 120 to 152 GHz, which indicates a wide receiving and reradiating bandwidth. The simulated isolation between Port 1 and Port 2 is greater than −17 dB within the frequency band from 121 to 147 GHz. Figure 3 shows the simulated results of normalized radiation patterns for the two polarizations at 135 GHz. The gains for the two polarizations are 6.32/6.8 dBi at 135 GHz, respectively.

## 3. Reflective Phasing Element

### 3.1. Design of the Reflective Phasing Unit Cell

**Figure 4:**
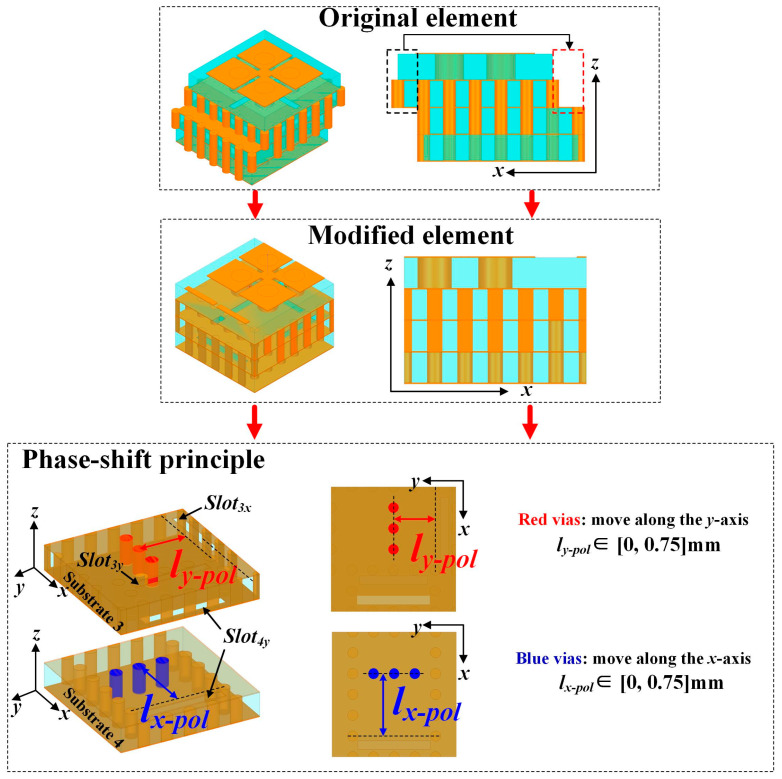
Configuration of the proposed dual-polarized reflective element with PBCs and its phase-shift principle.

As shown in Figure 4, the proposed reflective unit cell is based on a dual linear-polarized receiving/reradiating element connected by two different SIW phase-delay structures. The two rows of metallic vias in Substrates 3 and 4, marked in red and blue, respectively, represent the SIW short-end section for the two orthogonal polarizations. They effectively function as ground, reflecting and reradiating the energy received by the unit cell back to free space. By changing their positions, controlled by the parameters *l_x_pol_* and *l_y_pol_*, the phase responses for the two orthogonal polarizations can be independently manipulated. The reflection performance of the proposed element can be simulated by HFSS using Floquet ports and periodic boundary conditions (PBCs). The parameters *l_x-pol_* and *l_y-pol_* vary between 0 and 0.75 mm, with an increment of 0.05 mm.

### 3.2. Simulated Results of the Reflective Phasing Unit Cell

**Figure 5:**
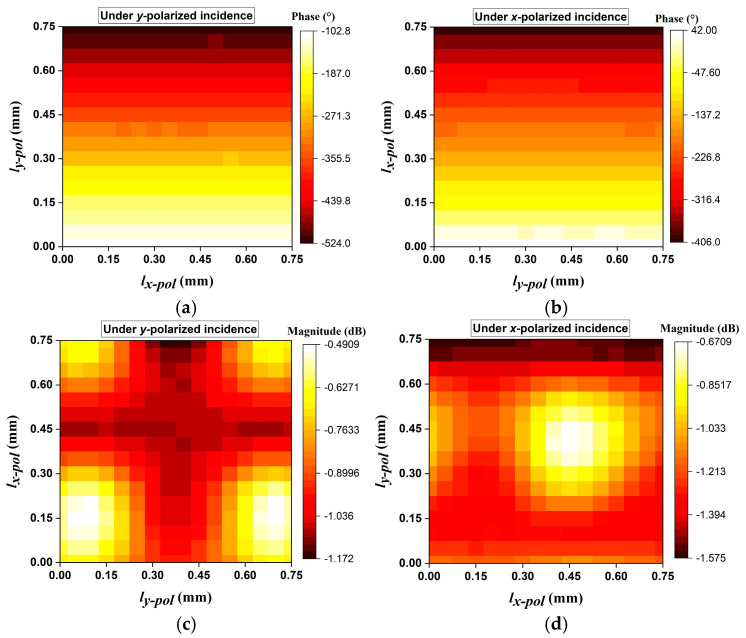
Simulated results of the proposed dual-polarized reflective element under normal incidence at 135 GHz. Reflection phase responses under (**a**) *y-*polarized and (**b**) *x-*polarized excitations. Reflection magnitude responses under (**c**) *y-*polarized and (**d**) *x-*polarized excitations.

The reflection phase responses under normal incidence are presented in Figure 5a,b. It can be observed that the proposed dual-polarized element can provide a phase-shifting range of over 400° for both the *x-* and *y-*polarized light. For the *y*-polarized illumination, the phase responses are mainly controlled by the parameter *l_y-pol_* rather than the parameter *l_x-pol_*; for the *x*-polarized illumination, the phase responses are mainly controlled by the parameter *l_x-po_*_l_ rather than the parameter *l_y-pol_*. Therefore, the phase responses for each polarization can be controlled independently. From Figure 1 and Figure 4, it can be seen that the length of the feeding path for the RA element operating under *x*-polarization is mostly longer than that of the feeding path for the RA element operating under *y*-polarization. Therefore, the loss of the RA element operating under *x*-polarization is a bit greater than that of the RA element operating under *y*-polarization. Figure 5c,d exhibit the simulated magnitude results at the center frequency of 135 GHz, which show that the reflection magnitudes for the *x-* and *y-*polarized incidences are larger than −1.2 dB and −1.6 dB, respectively. Additional simulations demonstrate that the proposed element maintains excellent characteristics, including a broad phase-shifting coverage and high reflection magnitude, across a wide bandwidth.

## 4. D-Band Reflectarray Antenna

### 4.1. Design of the Reflectarray Antenna

**Figure 6:**
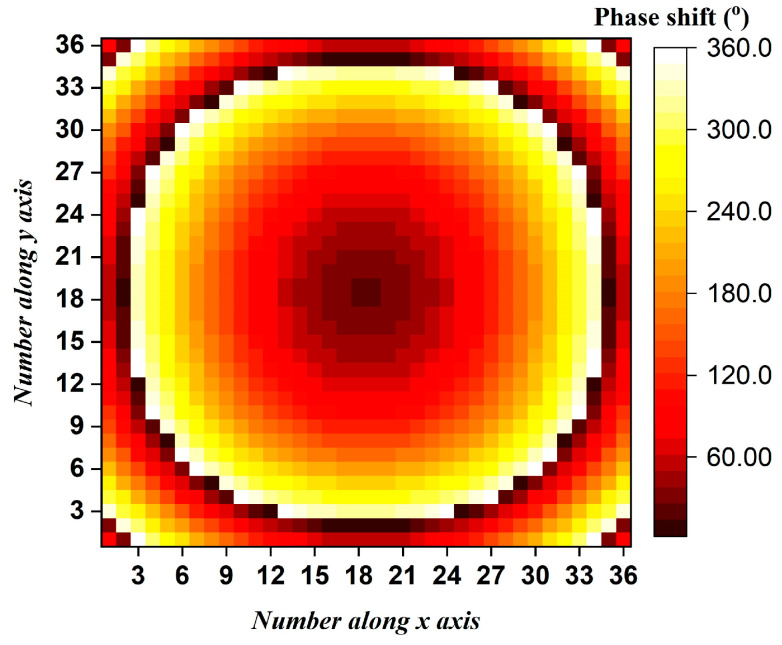
Required phase-shift distribution for both *x-* and *y-*polarized light.

Based on the proposed RA element, an RA prototype is designed, fabricated and measured for verification. The RA prototype has a square radiation aperture with 1296 elements. As shown in Figure 6, the required compensation phase value, *φ_mn_*, for each RA element can be calculated using the traditional ray-tracing method [3]:

(1) $$\varphi_{mn}=-k\left(r_{fmn}-\vec{u_{0}}\cdot {\vec{r}}_{mn}\right)+2\pi N,N=0,\pm 1,\pm 2,\cdot \cdot \cdot$$

where the parameter *k* represents the free-space wavenumber, the parameter *r_fmn_* is the spatial distance between the feed source and the *mn*th element, the parameter *u*_0_ represents the unit vector in the desired main beam direction, and the parameter *r_mn_* is the position vector of the *mn*th element.

### 4.2. Measurement Setup

**Figure 7:**
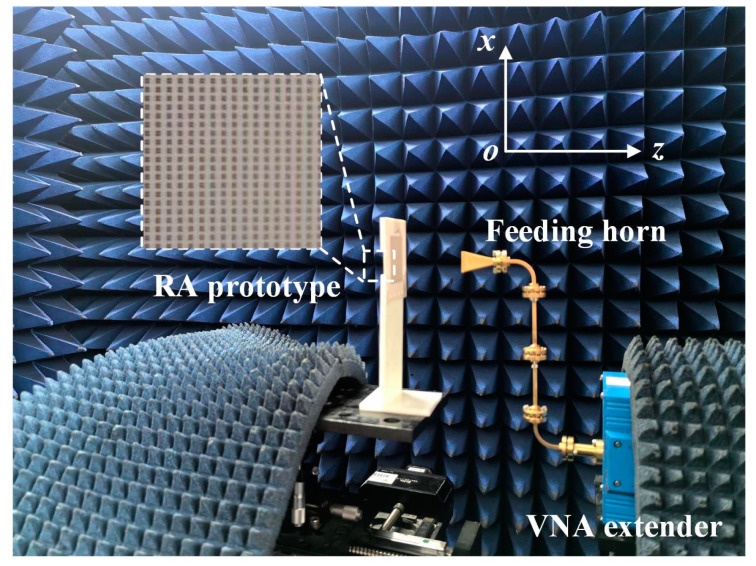
Photograph of the fabricated prototype and the measurement setup in a THz chamber.

Figure 7 presents the measurement setup of the proposed D-band RA antenna in a THz chamber, including a vector network analyzer (Agilent N5245A PNAX), a pair of OML WR-6 waveguide extenders, and an N5261A header controller. A standard horn antenna, manufactured by Millitech Corporation, is placed above the aperture center to feed this RA. The half-power beamwidth of the feeding horn antenna is 10.24° in the E-plane and 11.79° in the H-plane at 140 GHz. To provide proper illumination for the RA, the f/D ratio is chosen to be 2.26. Some D-band waveguide connectors, including 2-inch straight waveguides and 90° E/H-plane waveguide bends, are combined in the experiment to avoid the shielding effect caused by the OML extender. By carefully adjusting the relative position between the feeding horn and the RA prototype, radiation patterns can be obtained by rotating the rotary table.

### 4.3. Measured Results

**Figure 8:**
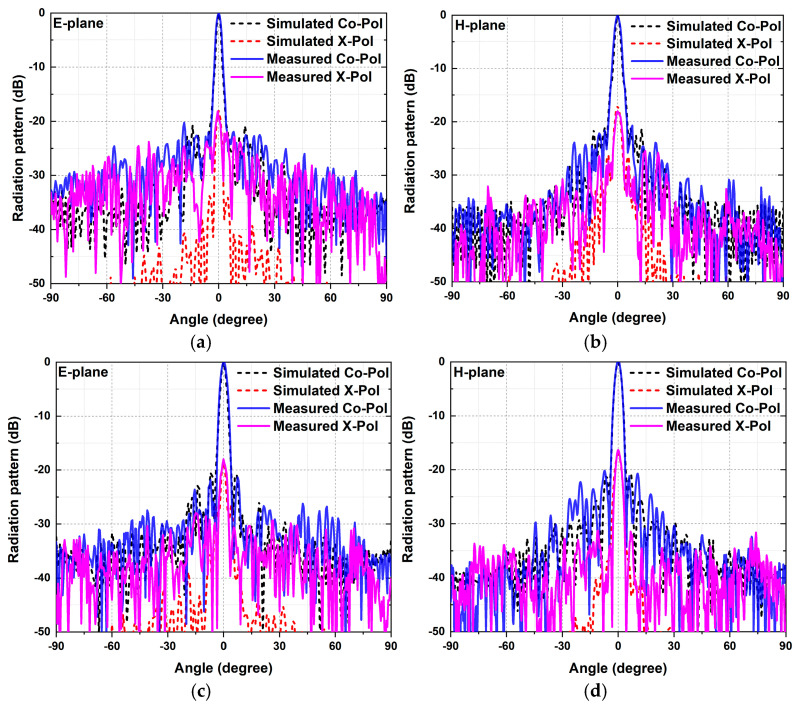
Simulated and measured radiation patterns at 135 GHz. (**a**) E-plane under *x-*polarized illumination. (**b**) H-plane under *x-*polarized illumination. (**c**) E-plane under *y-*polarized illumination. (**d**) H-plane under *x-*polarized illumination.

**Figure 9:**
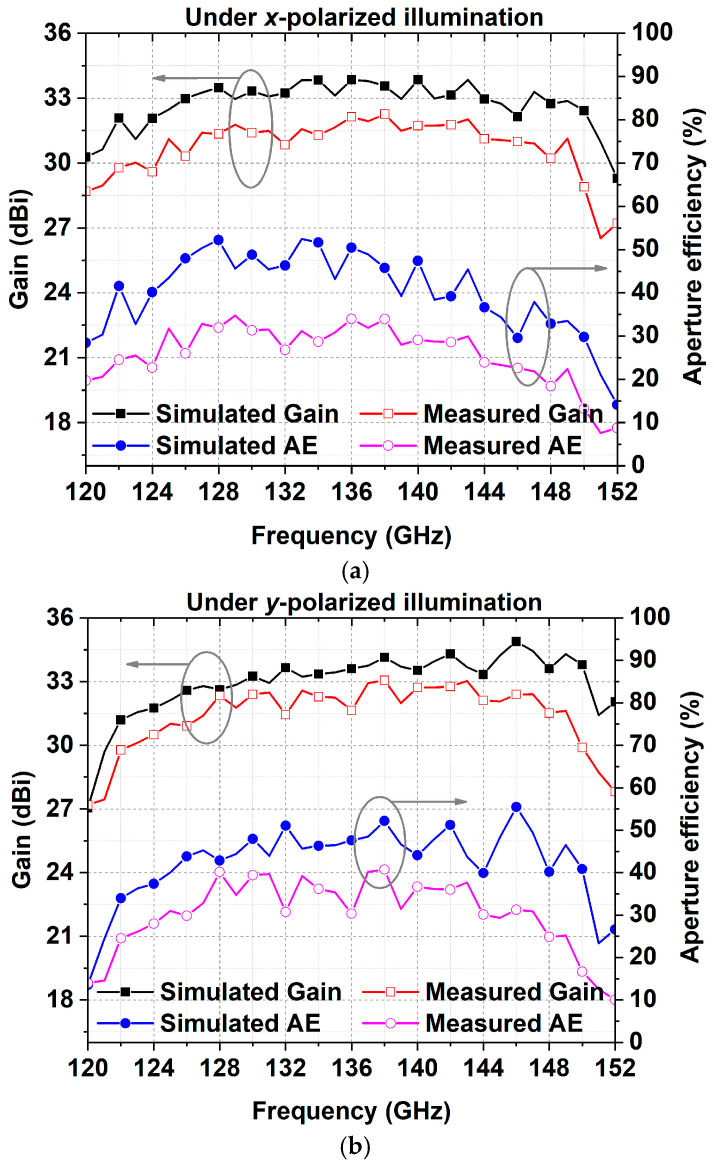
Gain and aperture efficiency of the proposed RA antenna: (**a**) under x-polarized illumination; (**b**) under y-polarized illumination.

As shown in Figure 8, the measured radiation patterns in the two principal planes under different polarizations are consistent with the simulated results. The measured 3 dB beamwidths in the E- and H-planes under *x-*polarized illumination are 2.3° and 2.6°. The E- and H-plane radiation patterns have 3 dB beamwidths of 2.5° and 2.4° when the incident waves are *y-*polarized. Figure 9 presents the simulated and measured gain and the aperture efficiency. The aperture efficiency can be calculated using the following equation:

(2) $$\eta_{a}=\frac{G\lambda_{0}^{2}}{4\pi A}$$

where the parameter *A* represents the physical area of the RA aperture, the parameter *G* is the antenna gain, and the parameter *λ*_0_ is the corresponding wavelength. At 135 GHz, the measured gains of the proposed RA prototype under *x-* and *y-*polarized illumination are 31.65 and 32.25 dBi, with corresponding aperture efficiencies of 30.81% and 35.38%, respectively. The measured 3 dB gain bandwidths for the *x-* and *y-*polarized excitations are 122–149 GHz (20%) and 123–149 GHz (19.3%), respectively. The measured aperture efficiency and gain declined compared to the values obtained through the simulation. These discrepancies are predominantly the result of three factors: dielectric loss, feed source excursion in the experiment, and the machining error of the prototype.

A table showing a comparison between this work and other related published studies is presented in Table 2. It can be observed that the proposed dual-polarized RA prototype based on LTCC technology exhibits the advantages of high gain, high aperture efficiency, and broad gain bandwidth for D-band applications.

## 5. Conclusions

From the literature review, it was found that very few studies have been reported for dual-polarized RA antennas operating at over 100 GHz. In this paper, a dual linear-polarized RA element for applications in D-band wideband high-gain RA antennas is proposed using LTCC technology. Based on the receiving/reradiating principle, the proposed element comprises a dual-polarized ME dipole and a multilayer slot-coupling SIW phase-delay structure. By independently adjusting the phase-delay structure’s length, the coverages of phase shifts for both orthogonal polarizations exceeded 360°. Then, an RA prototype was fabricated through LTCC technology and tested in a THz chamber. For the two orthogonal polarizations, the maximum gains of the proposed RA were 32.25 and 33.03 dBi, respectively. The measured 3 dB gain bandwidths for the two orthogonal polarizations were 122–149 GHz (20%) and 123–149 GHz (19.3%), respectively.

## Figures and Tables

**Table 1:** Geometries of the proposed receiving/reradiating ME-dipole element.

Parameter	Value (mm)	Parameter	Value (mm)	Parameter	Value (mm)
*l_cavity*	1.2	*w_cavity*	1.2	*p*	0.2
*d*	0.1	*l_patch*	0.925	*d_1_*	0.4
*d_feed*	0.112	*w_1_*	0.05	*l_1_*	0.382
*l_1x_*	1.05	*w_1x_*	0.08	*l_1y_*	1.05
*w_1y_*	0.08	*l_2x_*	0.65	*w_2x_*	0.08
*l*_*2y*_	0.65	*w*_*2y*_	0.08	*l*_*3y*_	0.7
*w*_*3y*_	0.085	*w_2_*	0.8	*h_c_*	0.008
*h_1_*	0.008	*h_2_*	0.2	*h_3_*	0.2
*h_4_*	0.2				

**Table 2:** Comparison between this work and other published RA antennas.

Reference Number	Polarization	Process	Element Type	*f*_0_ (GHz)	Peak Gain (dBi)	AE (%)	Gain Bandwidth (%)
[4]	Single linear	3D Printing	Dielectric cell	30	30.75	28.9	24.8 (1 dB)
[5]	Single linear	3D Printing	Dielectric cell	220	27.4	27.6	20.9 (1 dB)
[6]	Single linear	CNC	Metallic cell	12.5	32.5	-	8.3 (1 dB)
[7]	Single linear	PCB	Dielectric cell	13.5	30.2	45.6	18.1 (1 dB)
[8]	Single linear	CNC	Metallic cell	100	28	50.1	25 (3 dB)
[10]	Single linear	PCB	Stacked patch	32	32.55	-	19 (1 dB)
[11]	Single linear	PCB	Multi- resonance	12.5	33.9	67%	21.6 (AE > 40%)
[12]	Single linear	PCB	Multi- resonance	10	26.6	44.6	23.3 (1 dB)
[13]	Single linear	PCB	Multi- resonance	35	27.86	51.7	35.71 (3 dB)
[14]	Single linear	PCB	Sub- wavelength	10	28.2	56.5	18 (1.5 dB)
[16]	Single linear	PCB	Delay line	10	25.78	50	38.5 (3 dB)
[17]	Single linear	PCB	Delay line	30	24	48	7 (3 dB)
[18]	Single linear	PCB	Delay line	42.5	32.83	51.18	12.94 (3 dB)
[19]	Single linear	PCB	Delay line	20	27.51	43.8	100 (stable radiation pattern)
[20]	Dual linear	PCB	Multi- resonance	32	34.3	48.6	18 (1.5 dB)
[22]	Dual linear	PCB	Multi- resonance	11.95	28.82	52	27 (2 dB)
[23]	Dual linear	PCB	Stacked patch	15	24.5	37.4	15 (1 dB)
[24]	Single linear	LTCC	Heterogeneous Strategy	140	31.4	46.32	6.5 (1 dB)
**This work**	**Dual** **linear**	**LTCC**	**Delay line**	**135**	**33.03**	**35.38%**	**20 (3 dB)**

## Data Availability

All data are included within the manuscript.
